# Substrate Specificity, Inhibitor Selectivity and Structure-Function Relationships of Aldo-Keto Reductase 1B15: A Novel Human Retinaldehyde Reductase

**DOI:** 10.1371/journal.pone.0134506

**Published:** 2015-07-29

**Authors:** Joan Giménez-Dejoz, Michal H. Kolář, Francesc X. Ruiz, Isidro Crespo, Alexandra Cousido-Siah, Alberto Podjarny, Oleg A. Barski, Jindřich Fanfrlík, Xavier Parés, Jaume Farrés, Sergio Porté

**Affiliations:** 1 Department of Biochemistry and Molecular Biology, Faculty of Biosciences, Universitat Autònoma de Barcelona, Bellaterra, Barcelona, Spain; 2 Institute of Organic Chemistry and Biochemistry, Academy of Sciences of the Czech Republic, Prague, Czech Republic; 3 Institute of Neuroscience and Medicine (INM-9) and Institute for Advanced Simulation (IAS-5), Forschungszentrum Jülich GmbH, Jülich, Germany; 4 Department of Integrative Structural Biology, Institut de Génétique et de Biologie Moléculaire et Cellulaire-Centre de Biologie Intégrative, CNRS, INSERM, UdS, Illkirch CEDEX, France; 5 Diabetes and Obesity Center, School of Medicine, University of Louisville, Louisville, Kentucky, United States of America; University Paris Diderot-Paris 7, FRANCE

## Abstract

Human aldo-keto reductase 1B15 (AKR1B15) is a newly discovered enzyme which shares 92% amino acid sequence identity with AKR1B10. While AKR1B10 is a well characterized enzyme with high retinaldehyde reductase activity, involved in the development of several cancer types, the enzymatic activity and physiological role of AKR1B15 are still poorly known. Here, the purified recombinant enzyme has been subjected to substrate specificity characterization, kinetic analysis and inhibitor screening, combined with structural modeling. AKR1B15 is active towards a variety of carbonyl substrates, including retinoids, with lower *k*_cat_ and K_m_ values than AKR1B10. In contrast to AKR1B10, which strongly prefers all-*trans*-retinaldehyde, AKR1B15 exhibits superior catalytic efficiency with 9-*cis*-retinaldehyde, the best substrate found for this enzyme. With ketone and dicarbonyl substrates, AKR1B15 also shows higher catalytic activity than AKR1B10. Several typical AKR inhibitors do not significantly affect AKR1B15 activity. Amino acid substitutions clustered in loops A and C result in a smaller, more hydrophobic and more rigid active site in AKR1B15 compared with the AKR1B10 pocket, consistent with distinct substrate specificity and narrower inhibitor selectivity for AKR1B15.

## Introduction

Members of the aldo-keto reductase (AKR) superfamily share an (α/β)_8_ barrel fold and are mostly monomeric (~35 kDa) NAD(P)H-dependent enzymes catalyzing the reduction of a wide variety of endogenous carbonyl compounds such as carbohydrates, lipid aldehydes, prostaglandins, steroids and retinoids [1,2]. Several AKR enzymes act in phase-I drug metabolism by transforming some xenobiotics, are induced by nuclear factor erythroid 2-related factor 2 (Nrf2) under oxidative stress, and are involved in cancer chemoresistance [3,4]. In the human genome, fifteen *AKR* genes have been described which belong to three different gene families (*AKR1*, *AKR6* and *AKR7*). The *AKR1B* subfamily gene cluster, located in chromosome 7q33-35, includes the *AKR1B1*, *AKR1B10* and *AKR1B15* genes. A syntenic gene cluster with four *loci* has been described in rodent species, although gene orthologs can only be unambiguously assigned for *AKR1B1* [5,6].

The most studied enzyme of the AKR1B subfamily is AKR1B1 or aldose reductase, which reduces glucose to sorbitol under hyperglycemia and has been involved in the secondary complications of diabetic disease [7]. Another member, AKR1B10, is normally expressed in adrenal gland and small intestine, and induced in several types of cancer, such as non-small cell lung carcinoma and hepatoma [3]. Both enzymes have been proposed as promising oncogenic targets [8,9] and for this reason, along with the role of AKR1B1 in diabetic disease, they have been the subject of many studies in the search of selective and potent inhibitors [10–15]. Unlike other members of the subfamily, AKR1B10 is highly active in the reduction of all-*trans*-retinaldehyde [5]. The third gene in the *AKR1B* cluster, *AKR1B15*, has been predicted in the last decade as a result of high-throughput sequencing and annotation projects, i.e. human genome. Recently, *AKR1B15* has been demonstrated to be a functional gene with low expression restricted to placenta, testes and adipose tissues. The *AKR1B15* gene undergoes alternative splicing giving rise to two protein isoforms, designated as AKR1B15.1 and AKR1B15.2. The former is a 316-amino acid protein encoded by *AKR1B15-201 mRNA* (Ensembl database) and showing 92% amino acid sequence identity with AKR1B10, whereas AKR1B15.2 (*AKR1B15-001*, Ensembl) has a longer N-terminus not homologous to other AKRs, and does not exhibit enzymatic activity or nucleotide binding [16]. AKR1B15.1 (henceforth referred to in this manuscript as AKR1B15 for brevity) is localized in the mitochondrial fraction and the recombinant protein was purified and characterized, showing limited *in vitro* activity with steroids and acetoacetyl-CoA [16]. Previously, AKR1B15.1 had been expressed in the insoluble fraction of mammalian cells, showing low activity with d,l-glyceraldehyde and 4-nitrobenzaldehyde [6]. Similarly to *AKR1B10*, the *AKR1B15* gene was found to be up-regulated in the airway epithelium by smoking [17] and by exposure to sulforaphane, a known activator of the antioxidant response [18]. Interest in the *AKR1B15* gene has risen lately because some allelic variants have been linked to a mitochondrial oxidative phosphorylation disease [19], serous ovarian carcinoma [20] and increased longevity [21].

With the aim of further characterizing the enzymatic function of AKR1B15, we have performed enzyme kinetics of the purified recombinant protein with retinaldehyde isomers and other typical carbonyl substrates of AKR1B10. We have also conducted a screening against potential inhibitors using compounds previously described for AKR1B1 or AKR1B10. Finally, based on the crystallographic structure of the AKR1B10 complex with NADP^+^ and tolrestat, we have constructed a model of the AKR1B15 active-site pocket.

## Materials and Methods

### Bacterial strains, plasmids and reagents

*E*. *coli* BL21(DE3) strain was obtained from Novagen, while plasmids pBB540 and pBB542 (containing the chaperone-coding genes *grpE*, *clpB* and *dnaK*, *dnaJ*, *groESL*, respectively), were a kind gift from Dr. A. de Marco [22]. The pET-28a vector containing the cDNA coding for isoform 2 of AKR1B15 (**UniProt ID: C9JRZ8-2**) had been described by Salabei *et al*. [6]. Tolrestat and sorbinil were generously provided by Prof. T.G. Flynn and Pfizer, respectively, whereas JF0064 (2,2’,3,3’,5,5’,6,6’-octafluoro-4,4’-biphenyldiol) was obtained from Sigma-Aldrich. *Trans-*2-hexenal and 4-hydroxy-2-nonenal were commercially obtained from Cayman Chemical. All other reagents, including substrates, were purchased from Sigma-Aldrich unless otherwise indicated.

### Protein expression and purification

*E*. *coli* BL21(DE3) strain transformed with pET-28a/AKR1B15 was grown in 1 L of 2xYT medium in the presence of 33 μg/mL kanamycin, while *E*. *coli* BL21(DE3) containing pBB540, pBB542 and pET-28a/AKR1B15 was grown in 6 L of M9 minimal medium supplemented with 20% glucose as a carbon source, in the presence of 34 μg/mL chloramphenicol, 50 μg/mL spectinomycin and 33 μg/mL kanamycin. Protein expression was then induced by the addition of 1 mM IPTG (Apollo Scientific) and cells were further incubated for 4 h at 22°C. Cells were then pelleted and resuspended in ice-cold TBI buffer (150 mM NaCl, 10 mM Tris-HCl, 5 mM imidazole, pH 8.0) containing 1% (v/v) Triton X-100. In the case of the non-chaperone-expressing *E*. *coli* BL21(DE3) strain, the TBI buffer also contained 1% (w/v) sarkosyl. The protein was purified using a His-Trap HP nickel-charged chelating Sepharose Fast Flow (GE Healthcare) 5-mL column using an AKTA FPLC purification system. The column was washed with TBI buffer and the enzyme was eluted stepwise with 5, 60, 100 and 500 mM imidazole in TBI buffer. The enzyme fraction eluted with 100 mM imidazole was loaded onto a PD-10 column (Millipore), which removed imidazole and changed the buffer to storage buffer (200 mM potassium phosphate, pH 7.4, 5 mM EDTA, 5 mM DTT). Finally, the protein monomer was purified through gel filtration chromatography using a Superdex 75 10/300 GL column (GE Healthcare) equilibrated with the storage buffer. In the case of the protein expressed in the *E*. *coli* BL21(DE3) strain, in the absence of chaperones, the TBI and storage buffers contained 0.1% (w/v) sarkosyl throughout the purification procedure. AKR1B10 and AKR1B1 were expressed and purified as described previously [23].

### Fluorimetric and spectrophotometric assays

NADPH binding was analyzed by quenching of Trp intrinsic fluorescence of 0.5 μM protein, using a Cary Eclipse (Varian) fluorimeter, in 20 mM sodium phosphate, pH 7.0, at 25°C in a final volume of 1 mL. The excitation wavelength was 290 nm and the emission wavelength was monitored at 340 nm. AKR1B10 was used as a control. The dissociation constant (*K*_D_) values were calculated by nonlinear fitting of experimental data, using Grafit 5.0 (Eritacus Software), to the Morrison equation:

$$\Delta F=\Delta F_{max}\cdot \frac{\left[E\right]+\left[NADPH\right]+K_{D}-\sqrt{{\left(\left[E\right]+\left[NADPH\right]+K_{D}\right)}^{2}-4\cdot \left[E\right]\cdot \left[NADPH\right]}}{2\cdot \left[E\right]}$$

The activity towards aldehydes, with the exception of retinaldehydes, was analyzed spectrophotometrically following the decrease in the absorbance of the cofactor NADPH at 340 nm (ε_340_ = 6,220 M^−1^·cm^−1^) or at 365 nm in the case of cinnamaldehyde (ε_365_ = 3,510 M^−1^·cm^−1^) [24]. Activities were determined in 100 mM sodium phosphate, pH 7.0, at 25°C, using 0.2 mM NADPH in 0.2-cm path length cuvettes, with freshly prepared substrate solutions. One unit of activity is defined as the amount of enzyme required to transform 1 μmol of substrate per min at 25°C.

### HPLC enzymatic activity assay

Activity assays with retinoids were carried out using an HPLC-based methodology [25]. Briefly, retinaldehyde isomers were solubilized using glass tubes by a 10-min sonication at molar ratio 1:1 with fatty acid-free bovine serum albumin in 90 mM potassium phosphate, 40 mM potassium chloride, pH 7.4. The actual amount of solubilized retinoid was determined based on the corresponding molar absorption coefficient in aqueous solutions at the appropriate wavelength: ε_400_ = 29,500 M^−1^·cm^−1^ for all-*trans*-retinaldehyde and ε_367_ = 26,700 M^−1^·cm^−1^ for 9-*cis-*retinaldehyde [25]. For retinol isomers, which were used as standards of the reaction product, their concentration was determined in hexane using ε_325_ = 51,770 M^−1^·cm^−1^ for all-*trans*-retinol [26] and ε_325_ = 43,765 M^−1^·cm^−1^ for 9-*cis*-retinol [27]. The reactions were started by the addition of cofactor and carried out for 15 min at 37°C in a final volume of 0.5 mL. With the aim to measure the steady-state enzymatic activity, the concentration of enzyme was kept from 25- to 100-fold lower than that of the substrate for all the enzymatic assays. The reactions were stopped by the addition of 1 mL of cold methanol and after two rounds of extraction with hexane, retinoids were analyzed by HPLC as previously described [25]. All retinoid manipulations were performed under dim light.

### Determination of kinetic constants and inhibition screening

All compounds tested as inhibitors were dissolved in DMSO and assayed in a final concentration of 0.1% (v/v) DMSO using 6 mM d,l-glyceraldehyde as a substrate. Kinetic constant and IC_50_ (compound concentration that inhibits enzymatic activity by 50%) values were calculated by fitting the initial rates to the appropriate equation using Grafit 5.0 (Eritacus Software) and values were given as the mean ± standard error of three experiments. Standard error values were less than 20% of the mean values.

### Homology model and conformational ensembles

The structural model of *apo-*AKR1B15 was obtained from the AKR1B10 ternary complex crystallographic structure (**PDB ID: 1ZUA**), used as a template for homology modeling, by running the SCWRL program [28]. Because of the high sequence identity (92%) between the two proteins, the approach that keeps invariant the conformation of the conserved residues was adopted. The flexibility features of both AKR1B10 and AKR1B15 were studied by means of computer simulations.

The hydrogen atoms were added to the structural model of AKR1B15 as well as to the AKR1B10 crystal by the pdb2gmx tool of the GROMACS program package [29]. The *apo* as well as *holo* forms of both structures were studied as follows: the all-atom models were energy minimized employing the Amber99sb-ildn force field [30,31] for the protein, parameters of Holmberg *et al*. [32] for the cofactor, and Generalized-Born implicit solvent model with parameters of Hawkins *et al*. [33]. The minimized structures were used as the input for the tCONCOORD algorithm [34], which generates a set of independent conformations based on geometrical constraints [35]. It was designed to accurately capture the protein flexibility and the validity of the resulting conformational ensembles has been proven on a variety of proteins, including also AKR1B1 [34]. By means of tCONCOORD, we generated and analyzed the ensembles of 2500 conformations, which were subsequently used to calculate root mean square fluctuations (RMSF) of backbone atoms.

The energy minimized geometries (i.e. the starting ones for conformational sampling) were evaluated employing PROCHECK [36] and Verify 3D [37], which allow checking their stereochemical quality and calculating the percentage of conformations in favored regions obtained from the Ramachandran plots.

### AKR1B15 ternary complexes

Geometries of the ternary complexes were derived from the homology model of *holo*-AKR1B15, which was built by superimposition of the *apo* form and *holo-*AKR1B10 (**PDB ID: 1ZUA**), including the cofactor coordinates into *apo*-AKR1B15. Considering that all-*trans*-retinaldehyde and 9-*cis*-retinaldehyde are substrates, the distance between the oxygen of retinaldehyde and the catalytic residues (i.e. His111 and Tyr49) should be less than 3 Å for productive catalysis to occur. Under this assumption, the substrates were manually docked into the active site of *holo*-AKR1B15. Inhibitors were automatically docked using AutoDock 4.0 [38]. The inhibitor JF0064 coordinates were obtained from the PDB (**PDB ID 4ICC**). The target geometry was extracted from the energy minimized all-*trans*-retinaldehyde complex (see below). For docking, the target was kept rigid, while all the torsional bonds in JF0064, except for the conjugated double bonds, were free to move. The docking parameters were the same as described previously [39].

The hydrogen atoms were added to the ternary complex and the complex was energy minimized by adopting the PM6-D3H4 method [40] combined with the COSMO solvent model [41]. The residues, with at least one atom within 5 Å from any atom of the cofactor, substrate or inhibitor, were allowed to move and the rest was kept frozen but included in the Hamiltonian calculation. The PM6-D3H4 method, which has been developed to accurately describe non-covalent interactions in biomolecules, represents a well-established computational tool [42] and recently it has been used in the study of the inhibition of AKR1B1 [43]. The final pdb files of *holo*-AKR1B15 and the ternary complexes were validated with the QMEAN server [44], with the flag “ignoring the agreement terms”, recommended for proteins known to have the correct fold. The volume of the active-site pocket was measured by using the POVME algorithm [45], whereas PyMOL (v.1.3; Schrödinger) was used for figure drawing.

## Results and Discussion

### Expression and purification of recombinant human AKR1B15

**Fig 1:**
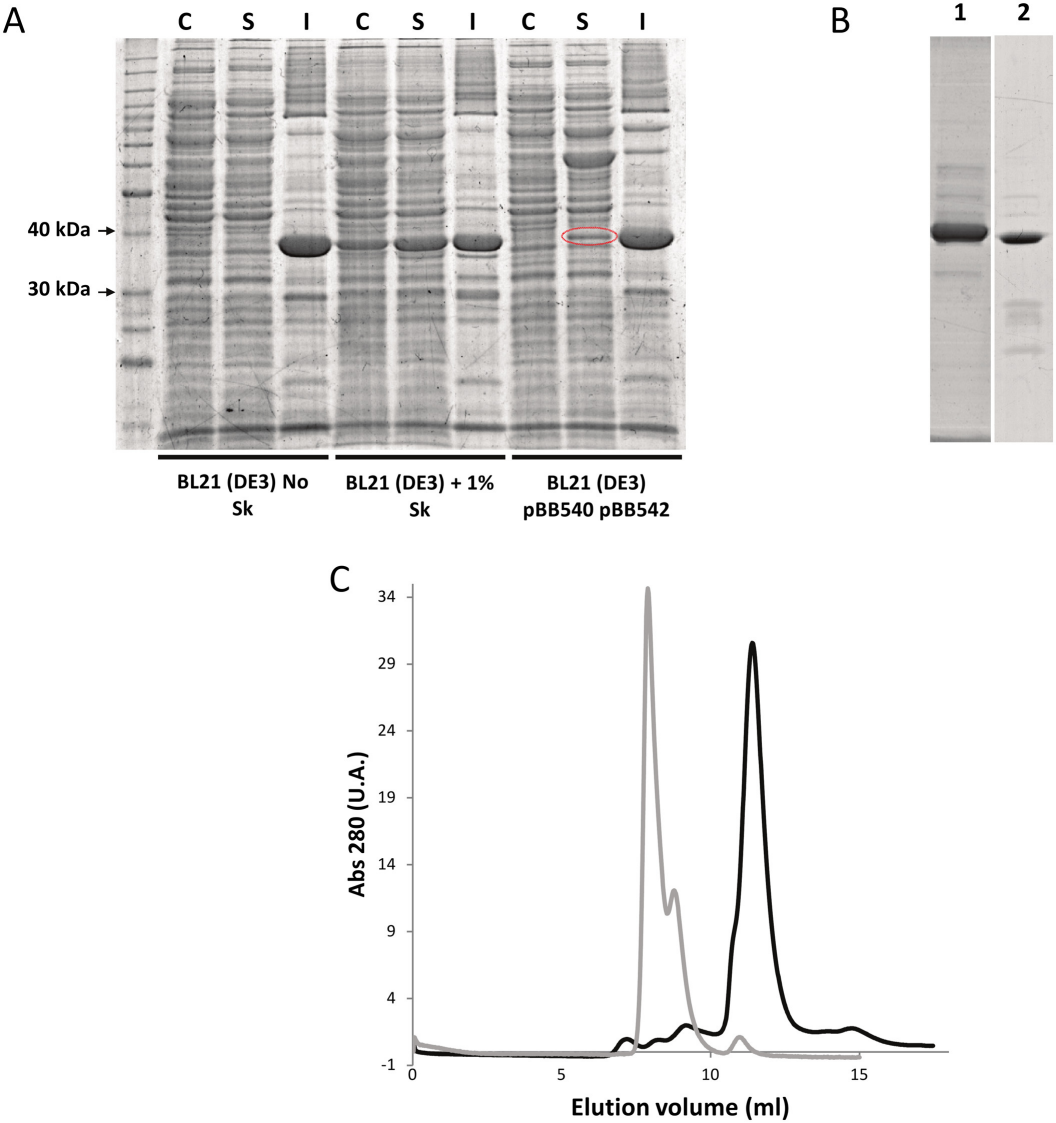
Expression and purification of recombinant human AKR1B15. (*A*) SDS-PAGE analysis of protein expression, showing that AKR1B15 was predominantly associated with the insoluble fraction of BL21(DE3) cell lysates. Treatment with 1% (w/v) sarkosyl (Sk) provided a much higher amount of AKR1B15 in the soluble fraction. In the case of BL21(DE3) pBB540 pBB542 cells, a protein band which is highlighted with a red oval was identified as human AKR1B15 by Peptide Mass Fingerprinting. Lanes: C, control for the soluble fraction not induced by IPTG; S, soluble fraction; and I, insoluble fraction. *(B)* SDS-PAGE analysis of protein purification, showing fractions eluted from the nickel affinity column chromatography using 100 mM imidazole. Lanes: 1, Protein eluted from the soluble fraction of BL21(DE3) + Sk; and 2, Protein eluted from the soluble fraction of BL21 (DE3) pBB540 pBB542. *(C)* Elution profile from the Superdex 75 10/300 GL column chromatography. AKR1B15 purified from soluble fraction of BL21(DE3) + Sk and from soluble fraction of BL21(DE3) pBB540 pBB542 are shown in grey and black lines, respectively. Major peaks eluting at 7.9 and 11.4 mL correspond to aggregated (132 kDa) and monomer (37 kDa) protein, respectively.

**Fig 2:**
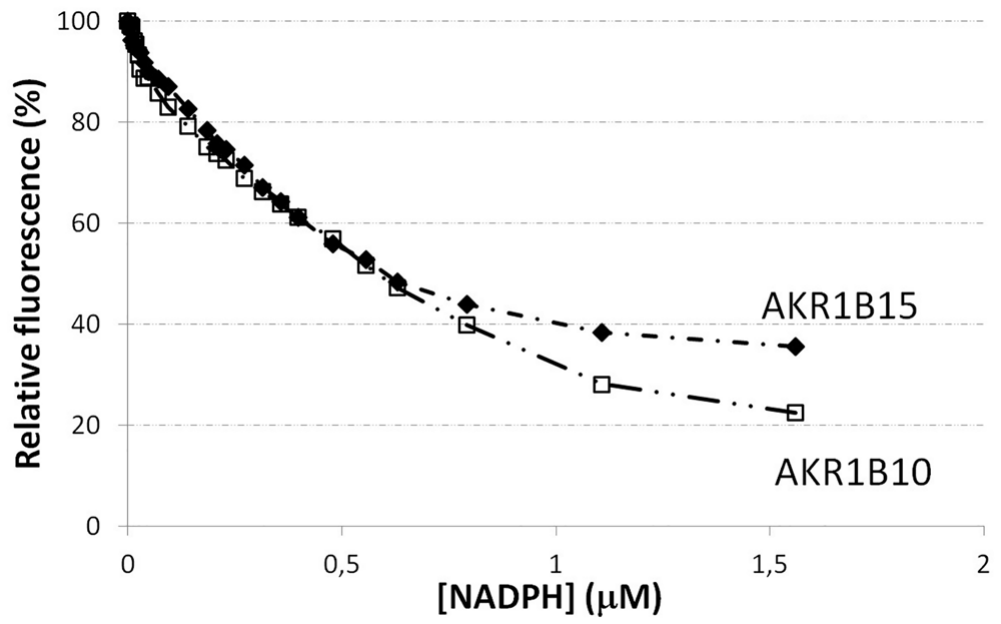
Quenching of AKR1B15 and AKR1B10 fluorescence upon binding of NADPH. Change of the protein fluorescence intensity (in percentage) upon addition of cofactor is shown. All proteins were used at a concentration of 0.5 μM in 20 mM sodium phosphate, pH 7.0, at 25°C. Graph symbols: AKR1B15 (diamonds), AKR1B10 (open squares).

We attempted the expression and purification of recombinant AKR1B15 using different *E*. *coli* strains and procedures. In all cases, the protein appeared to be mostly present in the insoluble fraction of cell lysates. Previously, Salabei *et al*. [6] had described a successful procedure based on the use of the anionic detergent sarkosyl (sodium *N*-lauroylsarcosinate). In our hands and in the presence of sarkosyl, the amount of soluble protein and the final yield were acceptable (Fig 1A and 1B). However, AKR1B15 was mainly associated with higher molecular weight aggregates (Fig 1C), which were found to be enzymatically inactive. Only a small fraction of AKR1B15 was found to be in an active monomeric form. A minimal concentration of sarkosyl (0.001%, w/v) was necessary to avoid protein precipitation. Sarkosyl had been described as an enzyme inhibitor [46], and was confirmed that it inhibited AKR1B15 following a non-competitive model (data not shown). As an alternative procedure for expression, an *E*. *coli* BL21(DE3) strain co-expressing three chaperone systems (DnaK-DnaJ-GrpE, ClpB and GroEL-GroES) [22] was used. Under these conditions, AKR1B15 was still found to be mainly associated with the insoluble fraction, but the amount of soluble protein recovered increased significantly (Fig 1A). Peptide mass fingerprint analysis of the soluble protein fraction separated by SDS-PAGE identified the isolated band as being human AKR1B15 (data not shown). The enzyme was purified with a final yield of 1 mg protein per liter of culture, when using minimal medium. Analysis by gel filtration chromatography showed a molecular weight of 37 kDa for the purified protein, suggesting that AKR1B15 was obtained as a monomer (Fig 1C). By analyzing the purified protein on SDS-PAGE (Fig 1B, lane 2), the major band corresponded to the 37-kDa enzyme, with some minor contaminating protein bands. Fluorescence analysis of cofactor binding allowed us to determine the *K*_D_ value for NADPH (113 ± 9 nM, Fig 2). The determined *K*_D_ value was in the same range with that of AKR1B10 (*K*_D_ = 92 ± 35 nM).

### Enzymatic activity and inhibition studies

It has been recently reported that AKR1B15 catalyzes the reduction of acetoacetyl-CoA and the carbonyl group at C17 position of sex steroids [16]. Additional results, obtained by using protein extracts from transfected human COS-7 cells, indicated that AKR1B15 exhibited very weak activity towards d,l-glyceraldehyde and 4-nitrobenzaldehyde [6], which are typical substrates of AKR enzymes. Our current analysis shows that the purified recombinant enzyme has broad substrate specificity and displays a significant enzymatic activity towards aliphatic and aromatic aldehydes and ketones (Table 1). AKR1B15 was active with d,l-glyceraldehyde, which was used as a substrate in the standard assay. The enzyme reduced 200 mM glucose with a much lower rate (12 mU/mg) than that exhibited by AKR1B1 (500 mU/mg), precluding the determination of kinetic constants. In contrast, AKR1B15 displayed a catalytic activity comparable or higher than that of AKR1B1 and AKR1B10 with a variety of physiological or model aldehydes and ketones of various classes. Among the compounds assayed, medium-chain (i.e. ≥ 6-carbon) aliphatic and aromatic carbonyl compounds were excellent substrates, with K_m_ values in the low micromolar range. Importantly, AKR1B15 was also active towards retinaldehyde isomers (Table 2), which along with acrolein, *trans*-2-hexenal, 4-hydroxy-2-nonenal and farnesal may constitute physiological substrates for this enzyme. The 9-*cis* isomer of retinaldehyde was the best substrate based on the catalytic efficiency (*k*_cat_/K_m_) due to a very low K_m_ value (160 nM) (Table 2), which could be determined by the use of an HPLC-based method providing higher sensitivity than the spectrophotometric assay. Regarding cofactor specificity, AKR1B15 was confirmed to be NADPH dependent (i.e. the enzymatic activity using 0.2 mM NADH was less than 5% of that with NADPH), as reported in [16], and described for most AKRs. The K_m_ value of AKR1B15 for NADPH (5 μM) is within the same range as those of AKR1B1 and AKR1B10 (Table 1).

**Table 1:** Kinetic constants of AKR1B15, AKR1B10 and AKR1B1 with aldehydes and ketones.

Substrate	AKR1B15			AKR1B10			AKR1B1		
	K_m_ (μM)	*k*_cat_ (min^−1^)	*k*_cat_/K_m_ (mM^−1^·min^−1^)	K_m_ (μM)	*k*_cat_ (min^−1^)	*k*_cat_/K_m_ (mM^−1^·min^−1^)	K_m_ (μM)	*k*_cat_ (min^−1^)	*k*_cat_/K_m_ (mM^−1^·min^−1^)
**Carbohydrate aldehydes**									
d,l-glyceraldehyde	880	10.7	12.4	6,000^a^	35^a^	6^a^	50^a^	31^a^	660^a^
**Aromatic aldehydes**									
pyridine-3-aldehyde	2.9	9	3,150	13^b^	150^b^	12,000^b^	10^b^	61^b^	6,100^b^
benzaldehyde	<1*	12.1	>12,100	9.7^b^	104^b^	11,000^b^	21^b^	91^b^	4,300^b^
cinnamaldehyde	<1*	13.3	>13,300	34.7	240	6,900	31	29	950
**Alkanals**									
hexanal	3.1	7.3	2,300	112^b^	142^b^	1,300^b^	5^b^	28^b^	5,600^b^
**Alkenals**									
acrolein	36	8.8	240	110^c^	116^c^	1,070^c^	884^c^	11^c^	12^c^
*trans*-2-hexenal	5	11.3	2,200	28^b^	49^b^	1,700^b^	9^b^	16^b^	1,800^b^
4-hydroxy-2-nonenal	2.2	5.2	2,500	31^c^	121^c^	3,900^c^	716^c^	50^c^	70^c^
citral	1.5	5.5	3,700	4.5	35	7,400	35	68	1,750
farnesal	<1*	4.8	>4,800	2.3^b^	30^b^	13,000^b^	37^b^	27^b^	700^b^
**Ketones**									
2-butanone	780	10.5	13.5		LA			LA	
3-buten-2-one	21.3	8.2	380		LA			LA	
3-nonen-2-one	1.7	6.76	4,000		LA			LA	
2-cyclohexen-1-one	365	4.41	12.1		LA			LA	
**α-Dicarbonyls**									
2,3-butanedione	<1*	10.6	>10,600	540^d^	260^d^	480^d^	110^d^	23^d^	210^d^
2,3-hexanedione	<1*	9.5	>9,500	51^b^	79^b^	1,500^b^	17^b^	49^b^	2,900^b^
**β-Dicarbonyls**									
2,4-pentanedione	40	2.2	55	58,900	8.6	0.15	8,100	16.7	2.2
3,5-heptanedione	1,300	5.3	3.9	>50,000	-	-	12,000	26	2.2
**Cofactor**									
NADPH	5.7			10			2.9^e^		

Enzymatic activity was measured spectrophotometrically. For the determination of the kinetic parameters for NADPH, 3 and 60 mM d,l-glyceraldehyde was used with AKR1B15 and AKR1B10, respectively. LA, low activity (≤ 10 mU/mg) was detected at saturating concentration of substrate for AKR1B15; ND, not determined.

*Because of very low K_m_ value, data are only approximate.

^a^Data from [23].

^b^Data from [66].

^c^Data from [67].

^d^Data from [68].

^e^Data from [69].

**Table 2:** Kinetic constants with retinaldehyde isomers.

Substrate and parameter	AKR1B15	AKR1B10^*a*^	AKR1B1^*a*^
All-*trans*-retinaldehyde			
K_m_ (μM)	1 ± 0.3	0.6 ± 0.1	1.1 ± 0.1
*k*_cat_ (min^−1^)	5.4 ± 0.5	27 ± 1	0.9 ± 0.1
*k*_cat_/K_m_ (mM^−1^·min^−1^)	5,300 ± 1,700	45,000 ± 7,600	1,300 ± 160
9-*cis*-retinaldehyde			
K_m_ (μM)	0.16 ± 0.03	0.7 ± 0.1	0.4 ± 0.1
*k*_cat_ (min^−1^)	3.8 ± 0.2	0.9 ± 0.1	0.7 ± 0.2
*k*_cat_/K_m_ (mM^−1^·min^−1^)	25,600 ± 5,300	1,300 ± 190	1,500 ± 170

Enzymatic activity was measured by using the HPLC-based method.

^a^Data from [23]

The comparison between AKR1B15 and the other human AKR1B enzymes reveals some relevant distinct kinetic features (Table 1). In particular, ketones and α-dicarbonyl compounds were good substrates for AKR1B15, showing higher activity and lower K_m_ values than AKR1B10 and AKR1B1. The β-dicarbonyl compound, 2,4-pentanedione, was also reduced by AKR1B15, consistent with the reported activity with acetoacetyl-CoA, which also has two carbonyl groups in a β-position [16]. For other substrates, AKR1B15 exhibited lower K_m_ values than AKR1B10, suggesting that AKR1B15 could play a role in aldehyde detoxification, similar to what has been suggested for other AKR1B enzymes [47]. AKR1B15 resembles AKR1B10 in having high activity with retinoids, in contrast to AKR1B1 (Table 2). A distinct feature of AKR1B15 is that it keeps similar *k*_cat_ values towards all assayed substrates, including retinoids, suggesting a common rate-limiting step. In comparison, the *k*_cat_ values of AKR1B1 with both retinaldehyde isomers and AKR1B10 with the 9-*cis* isomer are significantly lower than those for other substrates. This decrease in *k*_cat_ values had been interpreted before as a change in the rate-limiting step (from cofactor dissociation to a slower step) of AKR1B1 and AKR1B10 reactions with these retinoids [23]. This seems not to be the case for AKR1B15. Finally, the higher specificity of AKR1B15 for the 9-*cis* isomer is unique for the human AKR1Bs, and it matches those of AKR1C3 [48], which like AKR1B15 also has bulky Phe residues at positions 299 and 304 in its active site, and chicken AKR [49].

**Fig 3:**
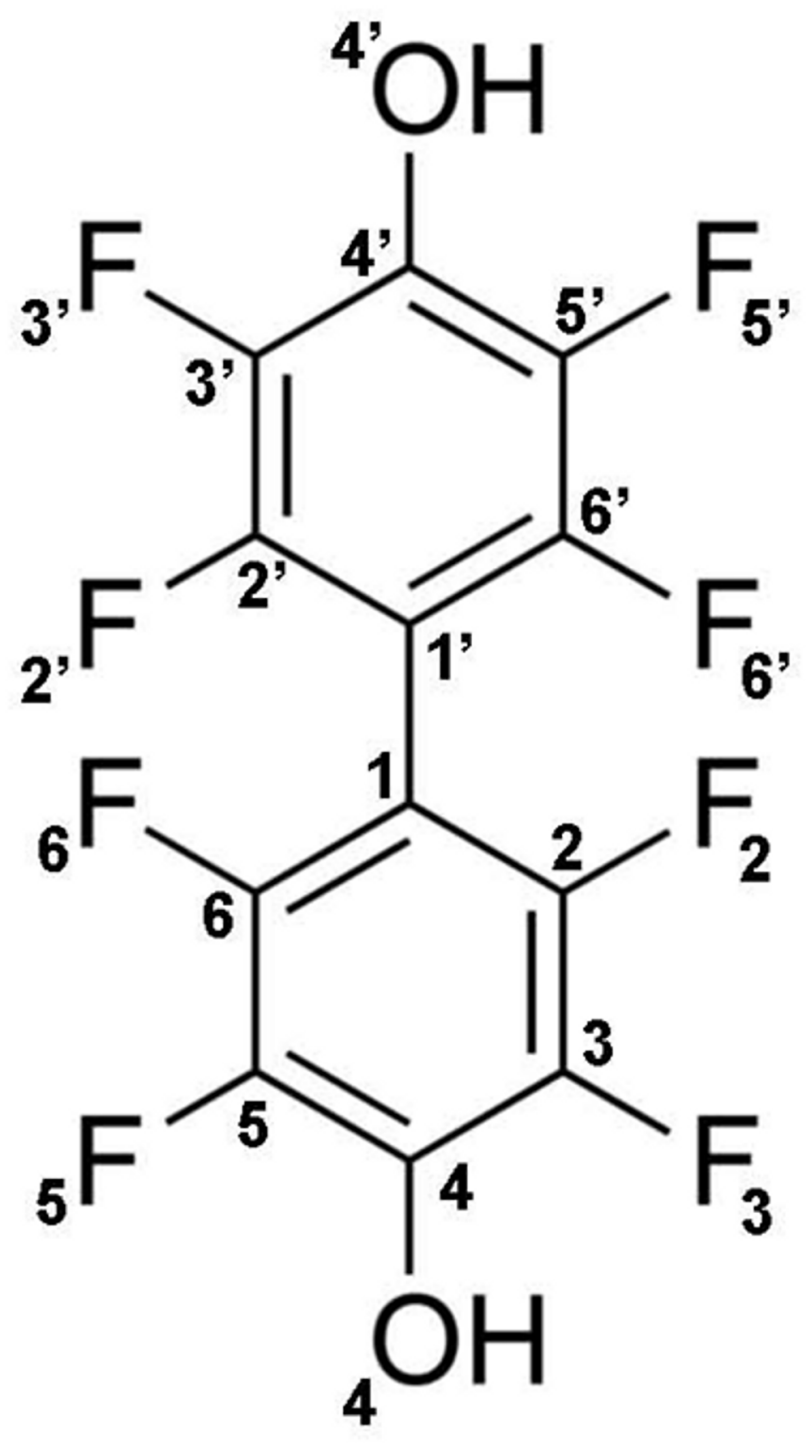
Molecular structure of compound JF0064, 2,2’,3,3’,5,5’,6,6’-octafluoro-4,4’-biphenyldiol.

Seven AKR inhibitors were tested against AKR1B15 using D,L-glyceraldehyde as substrate (Table 3). Among these inhibitors, there are five of the carboxylic acid type (tolrestat, epalrestat, oleanolic acid, sulindac and lithocholic acid), one of cyclic imide type (sorbinil) and one non-classical aldose reductase inhibitor or ARI (JF0064, which has been recently described by Cousido-Siah *et al* [15], Fig 3). Tolrestat, JF0064 and sulindac are potent inhibitors of AKR1B1 as well as of AKR1B10 (with IC_50_<10 μM for both enzymes). Sorbinil and epalrestat are more selective against AKR1B1 [50,51], while oleanolic and lithocholic acid are more selective against AKR1B10 [52]. For AKR1B15, only JF0064 showed a significant inhibition (IC_50_ = 0.034 ± 0.005 μM, Table 3), much stronger than for AKR1B1 and AKR1B10 [15]. The steroid lithocholic acid was found to be an inhibitor of AKR1B15, in agreement with the reported observation that certain steroids are good substrates for this enzyme [16].

**Table 3:** Inhibitory effect of different compounds on enzymatic activity.

IC_50_ (μM)			
Inhibitor	AKR1B15	AKR1B10	AKR1B1
Tolrestat	> 100	0.006	0.01^a^
Sorbinil	> 100	9.6^b^	0.55^b^
JF0064	0.034 ± 0.005	1.0^d^	0.3^d^
Epalrestat	> 50	0.33^c^	0.021^c^
Oleanolic acid	> 100	0.09^b^	124^b^
Sulindac	>100	0.35^c^	0.21^c^
Lithocholic acid	16.3 ± 7.6	0.12^d^	7.2^d^

The enzymatic activity assay with inhibitors was performed by using d,l-glyceraldehyde as a substrate. JF0064: 2,2’,3,3’,5,5’,6,6’-octafluoro-4,4’-biphenyldiol

^a^Data from [70].

^b^Data from [52].

^c^Data from [71].

^d^Data from [66]

### Structural model of AKR1B15

**Fig 4:**
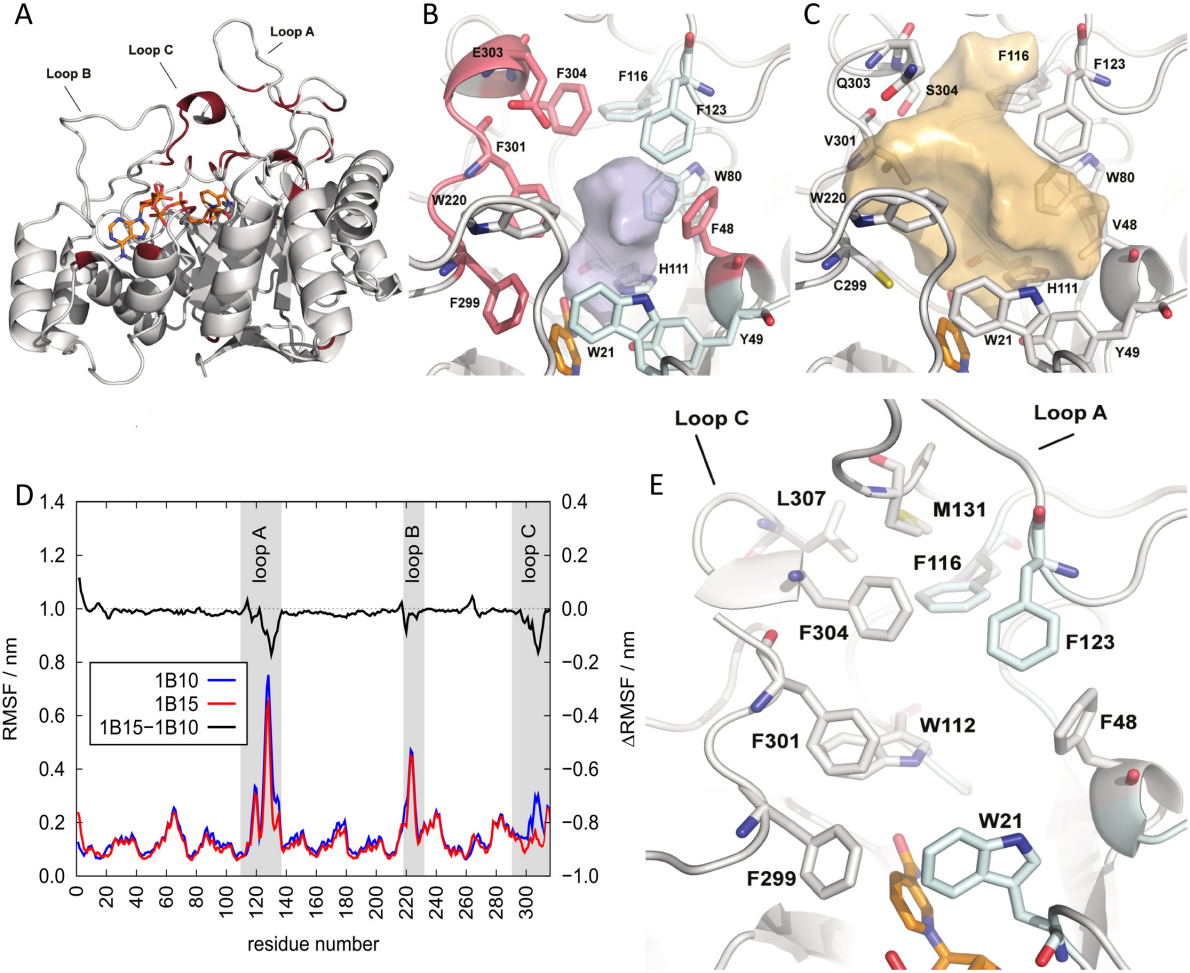
Model of AKR1B15 structure. (*A*) Side view of the (α/β)_8_ barrel. In red, the divergent residues between AKR1B15 and AKR1B10. (*B*) and (*C*) Active-site pockets of AKR1B15 and AKR1B10, respectively. The AKR1B15-specific residues are displayed in magenta. NADP^+^ cofactor is colored in orange. The surface contour of pockets is shown in grey and orange for AKR1B15 and AKR1B10, respectively. (*D*) The local conformational changes in *holo* forms of AKR1B15 (red line) and AKR1B10 (blue line) derived from computer simulations, as indicated by root mean square fluctuations (RMSF) of backbone atoms. The residues of loops A, B and C are highlighted by grey background. The difference in RMSF between the two enzymes is displayed as a black line in the top. *(E)* AKR1B15 loops A and C indicating potential contacts between different residues are shown as sticks, which may explain the low flexibility of the protein in this region. Figures have been drawn using PyMOL.

With the aim to compare the substrate-binding site of AKR1B15 with that of AKR1B10, and upon unsuccessful attempts of protein crystallization, a 3D homology model of AKR1B15 was built (Fig 4A). The SCWRL server was chosen, as it is designed specifically for predicting side-chain conformations, provided a fixed backbone usually obtained from an experimental structure. In practical terms, this is the case of AKR1B15, given its 92% sequence identity with AKR1B10. Model quality was checked by PROCHECK analyses, indicating that most residues are in the preferred regions (94.8%), whereas residues in allowed regions and outliers are 3.5 and 1.6%, respectively. The compatibility of the atomic model (3D) was checked with its own amino acid sequence (1D) using Verify 3D analysis. This analysis shows that there is no region with negative scores, which would otherwise indicate potential problems. In addition, the analysis using the QMEAN server indicates the reliability of the model, with a QMEAN score of 0.67 out of 1, and with all the Z-scores being consistent with the good quality of the structure. Thus it may be concluded that this model is suitable for structural studies.

### Model analysis

**Fig 5:**
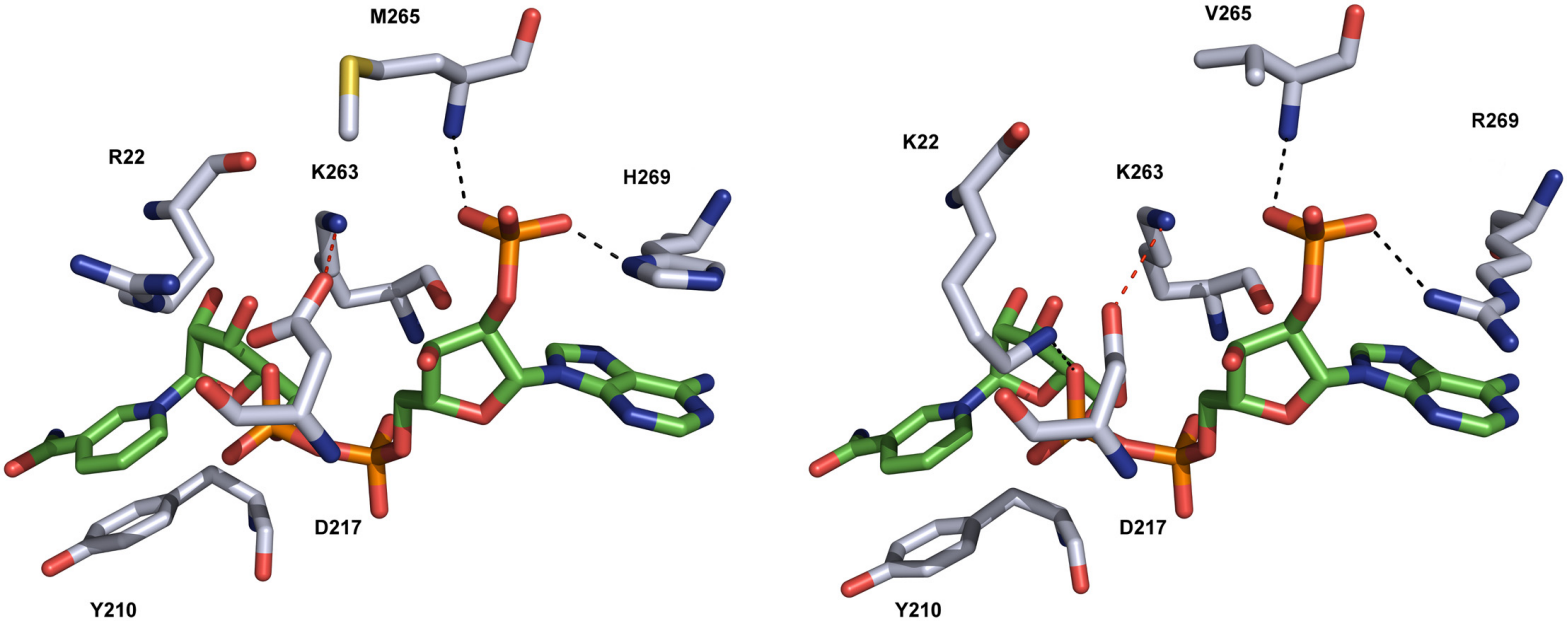
Comparison of the cofactor-binding site between the AKR1B15 model (*A*) and the AKR1B10 crystal structure (*B*). Interactions of Met265 and His269 with NADP^+^ in AKR1B15 are similar to those of Val265 and Arg269 in AKR1B10 (black dotted lines). His269 forms a π-stacking interaction with the adenine ring of the cofactor. The substitution of Lys22 by Arg in AKR1B15 prevents its interaction with the pyrophosphate bridge of NADP^+^. The salt bridge between Asp217 and Lys263 (red dotted line), acting as a safety belt in the coenzyme binding, and the π-stacking interaction of Tyr210 with the cofactor nicotinamide ring are conserved between the two AKRs. Carbon atoms of the cofactor are shown in green, whereas those of the enzyme are colored grey. Figures have been drawn using PyMOL.

Regarding the cofactor-binding site, AKR1B15 shares with AKR1B10 all residues, except for Arg22, Met265 and His269 (Fig 5). The change of Lys22 in AKR1B10 to Arg in AKR1B15 may prevent its interaction with the pyrophosphate bridge of NADP^+^ but, due to the mobility of the Arg side chain in solution, its interaction with the cofactor cannot be excluded. Met265 and His269 keep interactions with the same groups of NADP^+^ that involve Val265 and Arg269 in AKR1B10 (i.e. a hydrogen bond between the N atom of Met265 and the 2′-phosphate group, a second hydrogen bond between the Nε of His269 and the 2′-phosphate group, and a stacking interaction of His269 with the adenine ring). Interestingly, a His residue at position 269 and its interactions have also been described in the rat AKR1B14 X-ray structure [53]. The salt bridge between the side-chain of Asp217 and Lys263, acting as a safety belt in cofactor binding, and the stacking interaction of Tyr210 side chain with the nicotinamide ring are also conserved. This is consistent with the AKR1B15 cofactor preference for NADP(H) and the low K_m_ value of AKR1B15 for NADPH.

On the other hand, the active site displays high divergence between AKR1B15 and AKR1B10. The residue differences between the two proteins are concentrated in loops A and C which, along with loop B, give shape to the active-site pocket (Fig 4A). Thus, the catalytic residues (Asp44, Tyr49, Lys78 and His111) and those in loop B are strictly conserved. In contrast, Ser118, Leu122, Ala131, and Gly133 (in loop A), together with Cys299, Asn300, Val301, Leu302, Gln303, Ser304, and Tyr310 (in loop C) of AKR1B10 are substituted by Thr118, Phe122, Met131, Ser133, Phe299, Asp300, Phe301, Lys302, Glu303, Phe304 and Phe310, in AKR1B15. Noteworthy, residues Phe299, Phe301, Glu303 and Phe304, along with Phe48 (Val48 in AKR1B10), participate in the AKR1B15 active-site pocket, which is thus significantly smaller (60 Å^3^ for AKR1B15 *versus* 279 Å^3^ for AKR1B10) and more hydrophobic, compared to the AKR1B10 pocket (Fig 4B and 4C). Some of these substitutions might not only have a consequence on the shape, volume and hydrophobicity of the active site, but also on the flexibility of the polypeptide chain, which is most relevant in the loop regions. The analysis of conformational ensembles indicates that AKR1B15 would display less flexible loops A and C than AKR1B10 (Fig 4D). A detailed analysis of the AKR1B15 model shows van der Waals interactions between residues from these loops and residues from other protein regions (i.e. Trp21-Phe299_*loopC*, Phe48-Phe116_*loopA*, Phe48-Phe123_*loopA*, Phe48-Phe301_*loopA*, Trp112_*loopA*-Phe301_*loopC*, Phe116_*loopA*-Phe304_*loopC*, Phe123_*loopA*-Phe304_*loopC*, Met131_*loopA*-Phe304_*loopC* and Met131_*loopA*-Leu307_*loopC*). Such interactions result in a hydrophobic cluster (Fig 4E), which likely contributes to the lower flexibility of the AKR1B15 active site.

### Docking of substrates and the inhibitor JF0064

**Fig 6:**
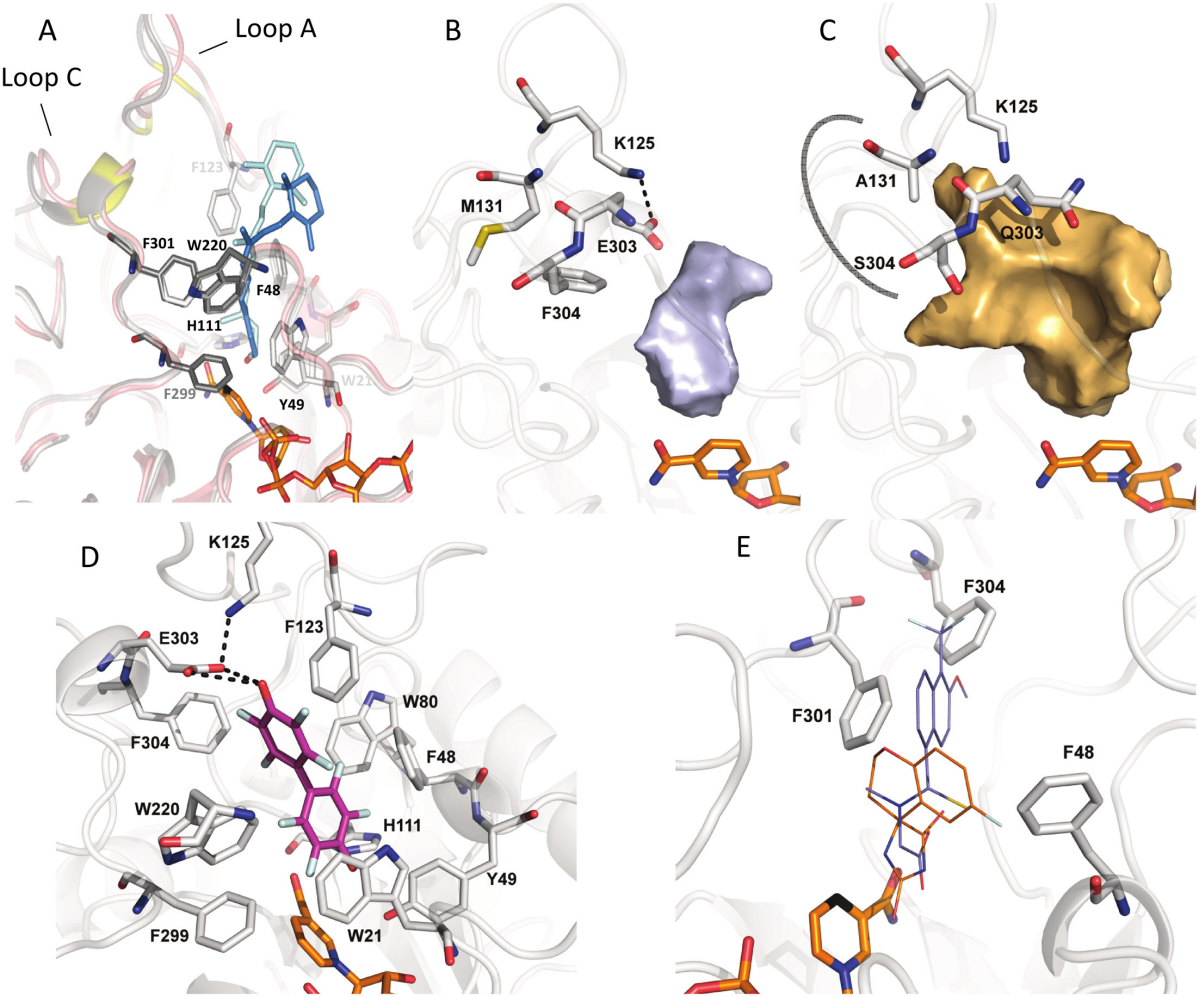
Molecular docking of substrates or inhibitors to the active-site pocket of AKR1B15. (*A*) Residues implicated in binding all-*trans*- and 9-*cis*-retinaldehyde are displayed in light and dark grey sticks; while the substrates are shown in light and dark blue, respectively. The residues found in the most external part of all-*trans*-retinaldehyde binding channel in AKR1B10 are highlighted in yellow. The energy minimized *apo*-conformation is displayed in magenta cartoon. (*B*) and (*C*) Side view of the surface contour of the active-site pocket, depicted in grey and orange for AKR1B15 and AKR1B10, respectively, to show the inhibitor “specificity pocket”. A thick grey curved line indicates the “specificity pocket” in AKR1B10. As it is shown, this pocket may not be opened in AKR1B15, likely due to the presence of bulky Phe residues. (*D*) The inhibitor JF0064 (PDB ID 4ICC) bound to AKR1B15 is displayed as sticks with C atoms in magenta, while residues interacting with the inhibitor are shown as sticks with C atoms in grey. (*E*) Steric hindrance preventing tolrestat (in blue) and sorbinil (in orange) from binding to the active site of AKR1B15. For this analysis, the AKR1B15 structure model was superimposed with the AKR1B10 crystallographic structures with tolrestat (PDB ID 1ZUA) and sorbinil (PDB ID 4GA8). NADP^+^ is colored in orange. Figures have been drawn using PyMOL.

As it has been described above, AKR1B15 is active towards retinoids, and thus the binding mode of all-*trans*- and 9-*cis*-retinaldehyde was analyzed. The obtained models were also analyzed with the QMEAN server and displayed similar scores (0.68/1 and 0.69/1, respectively) as the AKR1B15 holoenzyme model. The analysis showed that both substrates could be placed with their carbonyl groups at catalytic distance from the hydroxyl group of Tyr49, the Nε of His111, and the cofactor C4 atom (2.9, 2.9, and 3.2 Å, respectively). The two molecules would be positioned in a similar manner into a narrow and hydrophobic pocket, establishing contacts with Trp21, Phe48, Phe123, Trp220, and Phe301 (all-*trans*-retinaldehyde), and Phe48, Trp220, Phe299, and Phe301 (9-*cis*-retinaldehyde) (Fig 6A). A slight rearrangement of loop A and loop C (Fig 6A) could allow the establishment of a hydrogen-bond or an electrostatic interaction between Lys125 and Glu303 (Fig 6B).

The particular AKR1B15 topology brings the cyclohexene ring of all-*trans* and 9-*cis*-retinaldehyde further away from loops A and C than in AKR1B10 (Fig 6A, 6B and 6C). Thus these substrates do not interact with the residues that have been described as important for all-*trans*-retinaldehyde binding in AKR1B10, and which are in the most external part of the AKR1B10 pocket [23]. Notably, some of these residues are not interacting with the retinoid substrates in AKR1B15, i.e. Lys125 and Met131 (Ala in AKR1B10) from loop A, Glu303 (Gln in AKR1B10) and Phe304 (Ser in AKR1B10) from loop C. In AKR1B10, all-*trans*-retinaldehyde binding requires a rearrangement of Lys125, not necessary for 9-*cis*-retinaldehyde, explaining the higher *k*_cat_ value towards the former substrate [3]. In the case of AKR1B15, Lys125 is not involved in the binding of either substrate, being consistent with similar *k*_cat_ values. The hydrophobic pocket in the external region of the active site of the enzyme, where part of the polyene chain and the cyclohexene ring of the substrate bind, corresponds to the protein region which shows more rigidity in comparison to AKR1B10 (Fig 4D). Furthermore, this rigidity, likely due to the presence of the bulky Phe residues (Phe48, Phe299, Phe301 and Phe304), would make difficult the opening of the so-called “specificity pocket” (Fig 6B and 6C), which has been described for ARI binding in AKR1B10 and AKR1B1 and which usually accommodates the hydrophobic moiety of inhibitors [52,54]. This feature could have important functional consequences. For instance, it could explain the different substrate specificity and inhibitor selectivity of AKR1B15, since flexibility has been well established in connection with the active-site accessibility, and substrate and ligand binding [55].

The compound JF0064 is the only ARI found to significantly inhibit AKR1B15, probably due to the reduced volume of the active-site cleft and to the difficulty in opening the “specificity pocket”. In order to analyze JF0064 binding, a docking simulation along with energy minimization was performed. The model was again validated by using the QMEAN server and displayed a similar, though slightly better score than the rest of the AKR1B15 models (0.72/1). The Z-score (which is tending from negative digits to 0 when approximating to experimentally determined structures) of the AKR1B15-NADP^+^-JF0064 model (−0.92) is comparatively improved with respect to the AKR1B15 holoenzyme model (−1.64). All-*trans*- and 9-*cis*-retinaldehyde complexes show intermediate values of −1.51 and −1.35, respectively.

The conformation of the AKR1B15-NADP^+^-JF0064 complex corresponding to the energy minimum is displayed in Fig 6D. Binding would occur through van der Waals interactions with a large number of hydrophobic residues, and by establishing hydrogen bonds with catalytic residues (Tyr49 and His111) and Glu303. As it has been described above for substrates, the binding of the inhibitor also induced the same rearrangement of loop A and loop C, allowing for the interaction between Lys125 and Glu303. The other tested compounds did not inhibit AKR1B15 likely because of steric hindrance (e.g. Phe301 and Phe48 may clash against sorbinil, and Phe304, along with the fact that the “specificity pocket” could not be opened, may prevent tolrestat from binding) (Fig 6C and 6E).

### Physiological significance

The kcat/Km value of AKR1B15 for 9-*cis*-retinaldehyde (25,600 mM^−1^·min^−1^) is the highest among all the substrates checked so far, including the steroids and 3-keto-acyl-CoAs analyzed by Weber *et al*. [16], although these authors used different conditions for enzyme purification and kinetic studies. This is reminiscent of what has been observed for other members of the AKR superfamily, such as AKR1C3, which displays a high kcat/Km value for 9-*cis*-retinaldehyde but it is also active against steroids and prostaglandins [48]. Observations of dual substrate specificity have also been made for members of the short-chain dehydrogenase/reductase superfamily [56]. Thus, it is conceivable that AKR1B15 and some of these enzymes have a multifunctional role in the pre-receptor regulation of hormonal signaling pathways. The AKR1B15 specificity for the 9-*cis* isomer may suggest a major role in the control of RAR and RXR mediated signaling, but we cannot exclude other physiological functions. Regarding the reported localization of AKR1B15 in mitochondria, there is increasing evidence that retinoid metabolism takes place in different subcellular compartments [57,58], mitochondria being one of them. Carotenoids and their aldehyde metabolites can be generated by the asymmetric cleavage of β-carotene by β-carotene-9’,10’-oxygenase (BCO2), which is associated with the inner mitochondrial membrane [59]. Therefore, a putative physiological role of AKR1B15 in retinoid metabolism is compatible with its mitochondrial localization. The presence in mitochondria of other retinaldehyde reductases, such as RDH13 [57], gives further support to this notion. In addition, RDH10, an enzyme involved in retinoic acid synthesis, shifts between mitochondria associated membranes and lipid droplets during retinyl ester biosynthesis, similarly to cellular retinol-binding protein type 1 [60]. Retinol has also been pinpointed as a modulator of energy homeostasis in mitochondria by regulating oxidative phosphorylation [61]. An additional role of AKR1B15 in metabolizing lipid peroxidation products and alkenals in mitochondria cannot be ruled out [62,63].

## Conclusions

Despite its high sequence identity with AKR1B10, AKR1B15 appears to be an enzyme with a unique substrate specificity and narrower inhibitor selectivity. AKR1B15 displays distinct kinetic features with ketones, α-dicarbonyl compounds and is among the best 9-*cis*-retinaldehyde reductases within the AKR superfamily. Some of the most potent inhibitors of AKR1B1 and AKR1B10 did not inhibit AKR1B15. Amino acid substitutions clustered in residues located in loops A and C result in a smaller, more hydrophobic and more rigid active-site pocket of AKR1B15 as compared to that of AKR1B10. The structural model of AKR1B15 provides a powerful tool for the virtual screening of substrates and inhibitors for this enzyme. The distinct topology of the AKR1B15 fold should facilitate the design of more selective inhibitors, as it has been shown for other enzyme pairs with high sequence similarity [64,65]. Finally, the finding of all-*trans*- and 9-*cis*-retinaldehyde as substrates for AKR1B15 adds further complexity to the enzymatic pathways of retinoid transformations and their cross-talk with other hormonal signaling pathways, such as that of steroids. This opens a research line to elucidate the physiological contribution of this novel human retinaldehyde reductase.
